# Prognostic value of cardiovascular magnetic resonance in patients with strong suspicion for myocarditis

**DOI:** 10.1186/1532-429X-11-S1-P159

**Published:** 2009-01-28

**Authors:** Otavio R Coelho-Filho, Michael Jerosch-Herold, Sanjeev A Francis, Raymond Y Kwong

**Affiliations:** Brigham and Women's Hospital, Boston, MA USA

**Keywords:** Cardiovascular Magnetic Resonance, Myocarditis, Late Gadolinium Enhancement, Acute Myocarditis, Late Gadolinium Enhancement Imaging

## Background

While late gadolinium enhancement (LGE) imaging by Cardiovascular Magnetic Resonance (CMR) can detect evidence of myocarditis, the prognostic implication of this finding is still unknown.

## Purpose

The objective of this study is to assess the prognostic value of CMR in patients with suspected myocarditis.

## Methods

**Figure 1:**
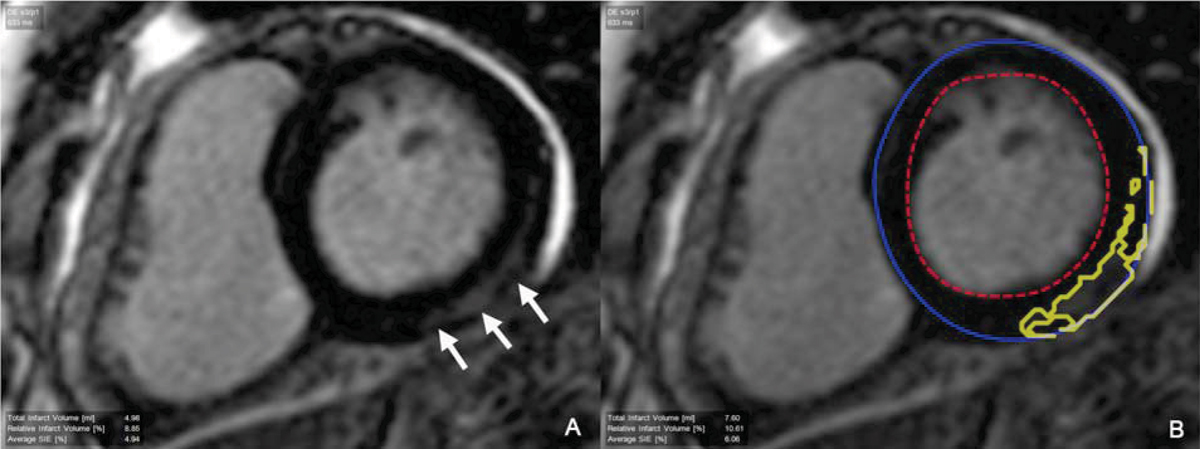
**An example of mid-wall LGE present in the mid-inferior and lateral walls (arrows) in a patient with a strong clinical suspicion of acute myocarditis (A)**. The area confined represents the detected LGE **(B)**.

We performed gadolinium-enhanced CMR in 76 patients (27 females, mean age 41 ± 14 years) with a strong clinical suspicion of myocarditis referred for CMR assessment. Patients were excluded if there was any clinical history of myocardial infarction or prior evidence of coronary artery disease. All enrolled patients had a recent normal coronary angiogram (n = 41) and/or a negative noninvasive stress test (n = 35). The CMR study was performed at a 1.5 Tesla scanner (GE Signa CVi) and a 3.0 Tesla scanner (Siemens Trio) and included short and long-axis function with cine SSFP imaging, and T1-weighted inversion recovery fast GRE imaging 10–15 minutes after a cumulative dose of 0.15 mmol/kg of gadolinium. In all CMR studies, we assessed the global and regional left ventricular function and presence of atypical (mid-wall or epicardial) LGE by CMR. The LGE mass was measured with a validated software (Pie Medical Imaging, CAAS, The Netherlands) using signal-intensity threshold (>3 SDs above remote myocardium) (Figure 1). In all patients, we obtained vital status and non-fatal MACE including heart failure admissions, significant ventricular arrhythmias requiring treatment, and recurrent myocarditis.

## Results

After a median follow-up period of 1.3 years (range 6 months to 48 months), only 7 patients experienced adverse events including 1 death, 2 heart failure admissions, 3 cases of significant ventricular arrhythmias, and 1 recurrent episode of acute myocarditis. Mean left ventricular ejection fraction of the study cohort was 54 ± 14%. LGE was detected in 23 patients (30.2%) and was not significantly associated with MACE by Cox regression analysis. Presence of LGE in this study cohort was associated with an elevated left ventricular mass (65 ± 17 vs. 75 ± 30 g/m^2^, p = 0.01), regional wall motion abnormalities (OR = 22, p = 0.03), and with peak serum troponin (2.4 ± 6 vs. 12 ± 9, p = 0.05). The relationship between LGE and elevated left ventricular mass persisted after adjusting for comorbidities such as hypertension and diabetes. The LGE mass (mean 3.5 ± 6.7 g) was associated with CK levels (r = 0.02, p = 0.02), with CK-MB levels (r = 0.41, p = 0.008) and with troponin levels (r = 0.48, p = 0.002).

## Conclusion

While a high proportion of patients were seen to have evidence of small myocardial involvement by LGE imaging consistent with acute myocarditis, overall patient prognosis of this clinical cohort with small LGE myocardial extent and preserved left ventricular ejection fraction is benign. CMR is helpful in the diagnosis of acute myocarditis and provides quantifiable parameters of the disease activity.

